# Supplementary material for: Nonequilibrium polysome dynamics promote chromosome segregation and its coupling to cell growth in Escherichia coli
Source: eLife. 2025 Jun 24;14:RP104276. doi: 10.7554/eLife.104276 (PMC12187137; doi:10.7554/eLife.104276)
Supplement: Supplementary file 1. [file elife-104276-supp1.docx]

**Supplementary file 1. Growth medium abbreviation and composition.**

| Abbreviation | Composition |
| --- | --- |
| Μ9acet | 1x M9 salts, 0.2% acetate (sodium salt) |
| M9acetCAAT | 1x M9 salts, 0.2% acetate, 0.1% casamino acids, 1μg/mL thiamine |
| Μ9fuc | 1x M9 salts, 0.2% fucose |
| Μ9fucCAAT | 1x M9 salts, 0.2% fucose, 0.1% casamino acids, 1μg/mL thiamine |
| M9fum | 1x M9 salts, 0.2% fumarate (disodium salt) |
| M9fumCAAT | 1x M9 salts, 0.2% fumarate, 0.1% casamino acids, 1μg/mL thiamine |
| M9GlcNAc | 1x M9 salts, 0.2% N-acetylglucosamine |
| M9GlcNAcCAAT | 1x M9 salts, 0.2% N-acetylglucosamine, 0.1% casamino acids, 1μg/mL thiamine |
| M9glu | 1x M9 salts, 0.2% glucose |
| M9gluCAAT | 1x M9 salts, 0.2% glucose, 0.1% casamino acids, 1μg/mL thiamine |
| M9gly | 1x M9 salts, 0.2% glycerol |
| M9glyCAAT | 1x M9 salts, 0.2% glycerol, 0.1% casamino acids, 1μg/mL thiamine |
| M9Lala | 1x M9 salts, 0.2% L-alanine |
| M9LalaCAAT | 1x M9 salts, 0.2% L-alanine, 0.1% casamino acids, 1μg/mL thiamine |
| M9Lara | 1x M9 salts, 0.2% L-arabinose |
| M9LaraCAAT | 1x M9 salts, 0.2% L-arabinose, 0.1% casamino acids, 1μg/mL thiamine |
| M9mala | 1x M9 salts, 0.2% malate (sodium salt) |
| M9malaCAAT | 1x M9 salts, 0.2% malate, 0.1% casamino acids, 1μg/mL thiamine |
| M9malt | 1x M9 salts, 0.2% maltose |
| M9maltCAAT | 1x M9 salts, 0.2% maltose, 0.1% casamino acids, 1μg/mL thiamine |
| M9mann | 1x M9 salts, 0.2% mannose |
| M9mannCAAT | 1x M9 salts, 0.2% mannose, 0,1% casamino acids, 1μg/mL thiamine |
| M9pyr | 1x M9 salts, 0.2% pyruvate (sodium salt) |
| M9pyrCAAT | 1x M9 salts, 0.2% pyruvate, 0,1% casamino acids, 1μg/mL thiamine |
| M9sorb | 1x M9 salts, 0.2% sorbitol |
| M9sorbCAAT | 1x M9 salts, 0.2% sorbitol, 0,1% casamino acids, 1μg/mL thiamine |
| M9succ | 1x M9 salts, 0.2% succinate (disodium salt) |
| M9succCAAT | 1x M9 salts, 0.2% succinate, 0,1% casamino acids, 1μg/mL thiamine |
| M9treh | 1x M9 salts, 0.2% trehalose |
| M9trehCAAT | 1x M9 salts, 0.2% trehalose, 0,1% casamino acids, 1μg/mL thiamine |
| M9xyl | 1x M9 salts, 0.2% xylose |
| M9xylCAAT | 1x M9 salts, 0.2% xylose, 0,1% casamino acids, 1μg/mL thiamine |
