# Supplementary material for: Nonequilibrium polysome dynamics promote chromosome segregation and its coupling to cell growth in Escherichia coli
Source: eLife. 2025 Jun 24;14:RP104276. doi: 10.7554/eLife.104276 (PMC12187137; doi:10.7554/eLife.104276)
Supplement: Supplementary file 2. [file elife-104276-supp2.docx]

**Supplementary file 2: *Escherichia coli* strains used in this study.**

| Strain | Genotype | Source |
| --- | --- | --- |
| MG1655 | *E. coli* MG1655 (F-lambda- *ilvG- rfb-50 rph-1*) | (Guyer et al., 1981; Jensen, 1993) |
| MG1655 (DE3) | *E. coli* MG1655 (DE3) | Kind gift from Dr K. Prather (Massachusetts Institute of Technology), (Tseng et al., 2010) |
| CJW5158 | *E. coli* BW25113 *hupA*::*hupA-mcherry-frt-kanR-frt* | (Gray et al., 2019) |
| CJW6723 | *E. coli* MG1655 Δ*lacZYA*::*P_lac_-egfp-μNS hup*A:*hupA-mcherry* | (Gray et al., 2019) |
| CJW6768 | *E. coli* MG1655 *rplA*::*rplA-meos2* | (Sanamrad et al., 2014) |
| CJW6769 | *E. coli* MG1655 *rspB*::*rpsB-meos2* | (Sanamrad et al., 2014) |
| CJW7019 | *E. coli* MG1655 *rplA*::*rplA-msfgfp-frt-kanR-frt* | (Gray et al., 2019) |
| CJW7020 | *E. coli* MG1655 *rplA*::*rplA-msfgfp* | (Gray et al., 2019) |
| CJW7021 | *E. coli* MG1655 *rpsB*::*rpsB-msfgfp* | (Gray et al., 2019) |
| CJW7144 | *E. coli* MG1655 Δ*lacZYA*::*P_lac_-mcherry-μNS-frt-kanR-frt* | This study |
| CJW7145 | *E. coli* MG1655 Δ*lacZYA*::*P_lac_-mcherry-μNS-frt-kanR-frt rplA*::*rplA-msfgfp* | This study |
| CJW7323 | *E. coli* MG1655 *rplA*::*rplA-msfgfp hupA*::*hupA-mcherry* | (Xiang et al., 2021) |
| CJW7466 | *E. coli* MG1655 (DE3) *hup*A:*hupA-mcherry* | This study |
| CJW7651 | *E. coli* MG1655 Δ*lacZYA*::*P_lac_-mcherry-μNS rplA*::*rplA-msfgfp* | This study |
| CJW7766 | *E. coli* MG1655 (DE3) *hup*A:*hupA-mcherry rplA*::*rplA-msfgfp-frt-kanR-frt* | This study |
| CJW7798 | *E. coli* MG1655 (DE3) *hup*A:*hupA-mcherry rplA*::*rplA-msfgfp-frt-kanR-frt* \| *pET28:mTagBFP2-CmR* | This study |
