# Supplementary material for: Nonequilibrium polysome dynamics promote chromosome segregation and its coupling to cell growth in Escherichia coli
Source: eLife. 2025 Jun 24;14:RP104276. doi: 10.7554/eLife.104276 (PMC12187137; doi:10.7554/eLife.104276)
Supplement: Supplementary file 3. [file elife-104276-supp3.docx]

**Supplementary file 3: Plasmids used in this study.**

| Plasmid name | Relevant genetic elements | Source |
| --- | --- | --- |
| pKD13 | *frt-kanR-frt-R6Kori-ampR* | (Datsenko and Wanner, 2000) |
| pKD46 | *araC-bet-exo-A101(Ts)ori-ampR* | (Datsenko and Wanner, 2000) |
| pCP20 | *cmR-A101(Ts)ori-ampR-flp-λ-repressor(Ts)* | (Datsenko and Wanner, 2000) |
| pER12 | *pBAD322A-gfp-μNS* | Kind gift from Dr A. Janakiraman (City College of New York), (Broering et al., 2005) |
| pER12-mcherry | *pBAD322A-mcherry-μNS* | This study |
| pAPG1 | *attB-pBAD322A-mcherry-μNS-attB-kanR-ColE1ori* | This study |
| pSB3C5-proA-B0032-E0051 | *PproA-lacZα-GFP-p15Aori-cmR* | (Davis et al., 2011) |
| pET28:GFP | *PT7-lacO-GFP-kanR-pMB1ori-lacI* | (Shis and Bennett, 2013) |
| pBAD:TagBFP2 | *araC-ParaBAD-mTagBFP2-ampR-pBAD322ori* | (Subach et al., 2011) |
| pET28:mTagBFP2 | *PT7-lacO-mTagBFP2-kanR-pMB1ori-lacI* | This study |
| pET28:mTagBFP2-CmR | *PT7-lacO-mTagBFP2-cmR-pMB1ori-lacI* | This study |
