# Supplementary material for: Nonequilibrium polysome dynamics promote chromosome segregation and its coupling to cell growth in Escherichia coli
Source: eLife. 2025 Jun 24;14:RP104276. doi: 10.7554/eLife.104276 (PMC12187137; doi:10.7554/eLife.104276)
Supplement: Supplementary file 4. [file elife-104276-supp4.docx]

**Supplementary file 4: DNA oligonucleotides used in this study.**

| Oligo name | Sequence | Source |
| --- | --- | --- |
| ER12-MCR-fwd | 5’ CTCCATACCCGTTTTTTTGGGCTAGCAGGAGGAATTCATGGTGAGCAAGGGCGAGGAG 3’ | This study |
| ER12-MCR-rev2 | 5’ CACTGGAGGAGCCTGCTTTTTTGTACAAACTTGTTGACTTGTACAGCTCGTCCATG 3’ | This study |
| μNSmCherry fwd | 5’ CACAGGTTGCTCCGGGCTATGAAATAGAAAAATGAATCCGTTGAAGCCTGATCGATGCATAATGTGCC 3’ | This study |
| μNSmCherry rev | 5’ AGCTCCAGCCTACACAGAGTTTGTAGAAACGCAAAAAG 3’ | This study |
| FRT_KanR fwd | 5’ GTTTCTACAAACTCTGTGTAGGCTGGAGCTGC 3’ | This study |
| FRT_KanR Rev | 5’ TTAAAGGTATTAAAAACAACTTTTTGTCTTTTTACCTTCCCGTTTCGCTC CTGTCAAACATGAGAATTAATTCC 3’ | This study |
| ColE1 fwd | 5’ GAGCGAAACGGGAAGGTAAAAAGACAAAAAGTTGTTTTTAATACCTTTAA CGTTCCACTGAGCGTC 3’ | This study |
| ColE1 rev | 5’ CAGGCTTCAACGGATTCATTTTTCTATTTCATAGCCCGGAGCAACCTGTG GGTTATCCACAGAATCAGG 3’ | This study |
| pAPG1 seq1 | 5’ GATCAAGCAGAGGCTGAAG 3’ | This study |
| pAPG1 seq2 | 5’ CCAGGCATCAAATTAAGC 3’ | This study |
| pAPG1 seq3 | 5’ TCTACGTGTTCCGCTTCC 3’ | This study |
| pAPG1 seq4 | 5’ TTGAAGCCTGATCGATGC 3’ | This study |
| pAPG1 seq5 | 5’ AAGATTAGCGGATCCTACC 3’ | This study |
| lacZYA_redμNS fwd | 5’ TATGTTGTGTGGAATTGTGAGCGGATAACAATTTCACACAGGAAACAGCT ATGGTGAGCAAGGGCGA 3’ | This study |
| lacZYA_redμNS rev | 5’ CAATTTTTATAATTTAAACTGACGATTCAACTTTATAATCTTTGAAATAA GGATCCGTCGACCTGCAG 3’ | This study |
| LacI fwd | 5’ GGCCGATTCATTAATGCAGCTGGC 3’ | This study |
| CynX rev | 5’ GGCCTGATAAGCGCAGCGTATC 3’ | This study |
| mCherry rev | 5’ GGTGCTTCACGTAGGCCTTGG 3’ | This study |
| KanR fwd | 5’ CGGAGAACCTGCGTGCAATCC 3’ | This study |
| mTagBFP2 fwd | 5’ CGGAGCTCGAATTCGGATCCTTAATTAAGCTTGTGCCCCAGTTTG 3’ | This study |
| mTagBFP2 fwd | 5’ CTTTAAGAAGGAGATATACCATGAGCGAGCTGATTAAGGAGAAC 3’ | This study |
| pET28_one fwd | 5’ TCCTTAATCAGCTCGCTCATGGTATATCTCCTTCTTAAAGTTAAAC 3’ | This study |
| pET28_one rev | 5’ GGATAACCGTATTACCGCCTTTGAGTGAGCTGATACCG 3’ | This study |
| pET28_two fwd | 5’ AGCGGTATCAGCTCACTCAAAGGCGGTAATACGGTTATCC 3’ | This study |
| pET28_two rev | 5’ TGGGGCACAAGCTTAATTAAGGATCCGAATTCGAGCTCC 3’ | This study |
| pET28mTagBFP2 fwd | 5’ CTTAGTGACTCGAATTCGCGCGCCAATCCGGATATAGTTCC 3’ | This study |
| pET28mTagBFP2 rev | 5’ ACTTTCTGGCTGGATGATGGACGTGAGTTTTCGTTCCACTG 3’ | This study |
| cmR fwd | 5’ AGTGGAACGAAAACTCACGTCCATCATCCAGCCAGAAAGTG 3’ | This study |
| cmR rev | 5’ GAACTATATCCGGATTGGCGCGCGAATTCGAGTCACTAAGG 3’ | This study |
