# Supplementary material for: Nonequilibrium polysome dynamics promote chromosome segregation and its coupling to cell growth in Escherichia coli
Source: eLife. 2025 Jun 24;14:RP104276. doi: 10.7554/eLife.104276 (PMC12187137; doi:10.7554/eLife.104276)
Supplement: Supplementary file 5. [file elife-104276-supp5.docx]

**Supplementary file 5: Chemicals used in this study.**

| Chemical | Source | Catalog number |
| --- | --- | --- |
| Agarose | AmericanBio | Cat#AB00972-00500 |
| 4’,6-diamidine-2′-phenylindole (DAPI), dihydrochloride fluorescent dye | Thermo Fisher Scientific | Cat#D1306 |
| Isopropyl β-D-1-thiogalactopyranoside (IPTG) | Sigma Aldrich | Cat#I5502 |
| Rifampicin | Sigma Aldrich | Cat#R3501 |
| Cephalexin | Sigma Aldrich | Cat#C4895 |
| A22 | Sigma Aldrich | Cat#SML0471 |
| Chloramphenicol | Sigma Aldrich | Cat#C0378 |
| 4’,6-Diamidine-2-phenylindole dihydrochloride (DAPI) | Thermo Fisher Scientific | Cat#D1306 |
| eBioscience^TM^ DRAQ5^TM^ | Thermo Fisher Scientific | Cat#65-0880-92 |
