# Supplementary material for: Nonequilibrium polysome dynamics promote chromosome segregation and its coupling to cell growth in Escherichia coli
Source: eLife. 2025 Jun 24;14:RP104276. doi: 10.7554/eLife.104276 (PMC12187137; doi:10.7554/eLife.104276)
Supplement: Supplementary file 6. [file elife-104276-supp6.docx]

**Supplementary file 6: Software used in this study.**

| Program / library / script | Package | Source |
| --- | --- | --- |
| Oufti | www.oufti.org | (Paintdakhi et al., 2016) |
| MATLAB | www.mathworks.com | Mathworks |
| Python | www.python.org | Python Software Foundation |
| Numpy | www.numpy.org | (Harris et al., 2020) |
| Scipy | www.scipy.org | (Virtanen et al., 2020) |
| Pytorch | www.pytorch.org | (Paszke et al., 2019) |
| Scikit-image | www.scikit-image.org | (Van Der Walt et al., 2014) |
| Scikit-learn | www.scikit-learn.org/stable/ | (Pedregosa et al., 2012) |
| Statsmodels | www.statsmodels.org/stable/index.html | (Seabold and Perktold, 2010) |
| Shapely | www.pypi.org/project/shapely/ | (Gillies, Sean et al., 2023) |
| Matplotlib | www.matplotlib.org | (Hunter, 2007) |
| Seaborn | www.seaborn.pydata.org | (Waskom, 2021) |
| Pandas | www.pandas.pydata.org | (McKinney, 2010) |
| Python | Omnipose neural network | (Cutler et al., 2022) |
| MATLAB | SuperSegger | (Stylianidou et al., 2016) |
| Python | Unet neural network | (Wiktor et al., 2021; Zhou et al., 2020) |
| snapshots_analysis_UNET_ghv.py | Snapshot image analysis (from UNET masks) – custom Python class | This study  ([www.github.com/JacobsWagnerLab/published/tree/master/Papagiannakis_2025](https://github.com/JacobsWagnerLab/published/tree/master/Papagiannakis_2025)) |
| snapshots_analysis_OUFTI_GrayGovers_ghv.py | Snapshot image analysis (from Oufti masks) – custom Python class | This study  ([www.github.com/JacobsWagnerLab/published/tree/master/Papagiannakis_2025](https://github.com/JacobsWagnerLab/published/tree/master/Papagiannakis_2025)) |
| snapshots_analysis_functions.py | Extraction of fluorescence and morphology statistics from cell snapshots – custom Python functions | This study  ([www.github.com/JacobsWagnerLab/published/tree/master/Papagiannakis_2025](https://github.com/JacobsWagnerLab/published/tree/master/Papagiannakis_2025)) |
| microfluidics_segmentation_ghv.py | Cell segmentation and tracking from time-lapse images in microfluidics – custom Python class | This study  ([www.github.com/JacobsWagnerLab/published/tree/master/Papagiannakis_2025](https://github.com/JacobsWagnerLab/published/tree/master/Papagiannakis_2025)) |
| microfluidics_analysis_functions_ghv.py | Extraction of fluorescence and morphology statistics from time-lapse images in microfluidics – custom Python functions | This study  ([www.github.com/JacobsWagnerLab/published/tree/master/Papagiannakis_2025](https://github.com/JacobsWagnerLab/published/tree/master/Papagiannakis_2025)) |
| Omnipose_to_python_ghv.py | Extraction of segmentation masks and tracked lineages from Omnipose/SuperSegger to Python for further analysis | This study  ([www.github.com/JacobsWagnerLab/published/tree/master/Papagiannakis_2025](https://github.com/JacobsWagnerLab/published/tree/master/Papagiannakis_2025)) |
| Otsu_phase_segmentation_ghv.py | Cell segmentation and tracking using Otsu thresholding (Otsu, 1979) | This study  ([www.github.com/JacobsWagnerLab/published/tree/master/Papagiannakis_2025](https://github.com/JacobsWagnerLab/published/tree/master/Papagiannakis_2025)) |
| LoG_adaptive_image_filter.py | Image filter that applies relative and local thresholding to segment fluorescence objects and particles | This study  ([www.github.com/JacobsWagnerLab/published/tree/master/Papagiannakis_2025](https://github.com/JacobsWagnerLab/published/tree/master/Papagiannakis_2025)) |
| Microfluidics_segmentation_and_tracking_example.pdf | Example for cell segmentation and tracking in microfluidics | This study  ([www.github.com/JacobsWagnerLab/published/tree/master/Papagiannakis_2025](https://github.com/JacobsWagnerLab/published/tree/master/Papagiannakis_2025)) |
| Analysis_of_cell_morphology_and_fluorescence_in_microfluidics.pdf | Example for the extraction of fluorescence and morphology statistics from time-lapse images in microfluidics | This study  ([www.github.com/JacobsWagnerLab/published/tree/master/Papagiannakis_2025](https://github.com/JacobsWagnerLab/published/tree/master/Papagiannakis_2025)) |
| Analysis_of_cell_morphology_and_fluorescence_in_agarose_pads.pdf | Example for the extraction of fluorescence and morphology statistics from cell snapshots | This study  ([www.github.com/JacobsWagnerLab/published/tree/master/Papagiannakis_2025](https://github.com/JacobsWagnerLab/published/tree/master/Papagiannakis_2025)) |
| Example_of_using_Otsu_based_segmentation_ghv.pdf | Example for the segmentation of antibiotic-treated cells using the Otsu threshold | This study  ([www.github.com/JacobsWagnerLab/published/tree/master/Papagiannakis_2025](https://github.com/JacobsWagnerLab/published/tree/master/Papagiannakis_2025)) |
| tune_diffusion.ipynb | Simulations of the minimal reaction-diffusion model that describes nucleoid segregation in *E. coli*. | This study  (https://github.com/qiweiyuu/polysome) |
| Time_lapse_on_agarose_pad | Python repository that includes the *fluorescence_analysis* class and its associated functions, used to track cell trajectories and analyze their fluorescence statistics in time-lapse images on agarose pads. | This study  (https://github.com/alexSysBio/Time_lapse_on_agarose_pad) |
| flowio_to_pandas | Python repository that includes the *flow_cytometry_class* class and its associated functions, used to parse FCS files into Pandas. This class can also be used for polygon and histogram gating. | This study  (https://github.com/alexSysBio/flowio_to_pandas) |
| nd2_to_array.py | Function used to parse .nd2 files into numpy arrays. | This study  (https://github.com/alexSysBio/Adding_ND2_images_to_python) |
| Bivariate_medial_axis_estimation.py | Functions used to draw the medial axis in segmented cell masks from snapshots or time-lapse images on agarose pads. | This study  (https://github.com/alexSysBio/Cell_medial_axis_definitions) |
| Unever_background_correction.py | Functions used to subtract the background from agarose-pad images. | This study  (https://github.com/alexSysBio/Image_background_subtraction) |
